# Early-Onset Epileptic Encephalopathy Responsive to Phenytoin: A Diagnostic Clue for Fibroblast Growth Factor 12 Mutation

**DOI:** 10.7759/cureus.53906

**Published:** 2024-02-09

**Authors:** Nadia M Saleem, Nidheesh Chencheri, Sen Thomas, Gail Alexander, Biju Madathil

**Affiliations:** 1 Department of Medicine and Surgery, Dubai Academic Health Corporation, Dubai, ARE; 2 Department of Pediatric Neurology, Al Jalila Children's Specialty Hospital, Dubai, ARE; 3 Department of Pediatric Emergency Medicine, Al Jalila Children's Specialty Hospital, Dubai, ARE; 4 Department of Neonatology, NMC Royal Women's Hospital, Abu Dhabi, ARE

**Keywords:** phenytoin, electroencephalogram spikes, cerebral atrophy, refractory seizures, developmental delay, fgf12 gene mutation

## Abstract

We present a case of a three-year-old girl with a rare genetic epilepsy with developmental delay. She was born to a non-consanguineous parentage and required resuscitation soon after delivery via cesarean section. The patient had her first seizure within 36 hours of life, which progressed into refractory epilepsy. She required multiple hospital admissions due to prolonged seizures. Despite being tried on multiple drug combinations over the years, she responded only to phenytoin. Basic imaging and other investigations, including genetic analysis, revealed a fibroblast growth factor 12 (FGF12) mutation. Mutations in these genes cause refractory early-onset seizures associated with severe developmental delay. Due to early and appropriate intervention with phenytoin, she had good seizure control which probably resulted in a better developmental outcome.

## Introduction

Fibroblast growth factor 12 (FGF12) mutations are extremely rare, with only a handful of cases reported worldwide. It has many roles in the body but is especially important in properly functioning the central nervous system [1,2]. Mutations in these genes cause a toxic gain of function of FGF12, assumed to lead to increased neuronal excitability. The typical phenotype includes moderate to mostly severe developmental delay /intellectual disability, and early‐onset drug‐resistant epilepsy with frequent status epilepticus. Some patients can have other neurological symptoms such as cerebellar ataxia, significant hypotonia, and feeding difficulties. FGF12 encodes for one of a family of four proteins that indirectly modulate the activities of voltage‐gated sodium channels (Nav). Mutations in voltage‐gated sodium channels are well‐known causes of epilepsy and epileptic encephalopathy [3]. A favorable response to the sodium blockers, especially phenytoin was reported in several cases.

## Case presentation

**Figure 1:**
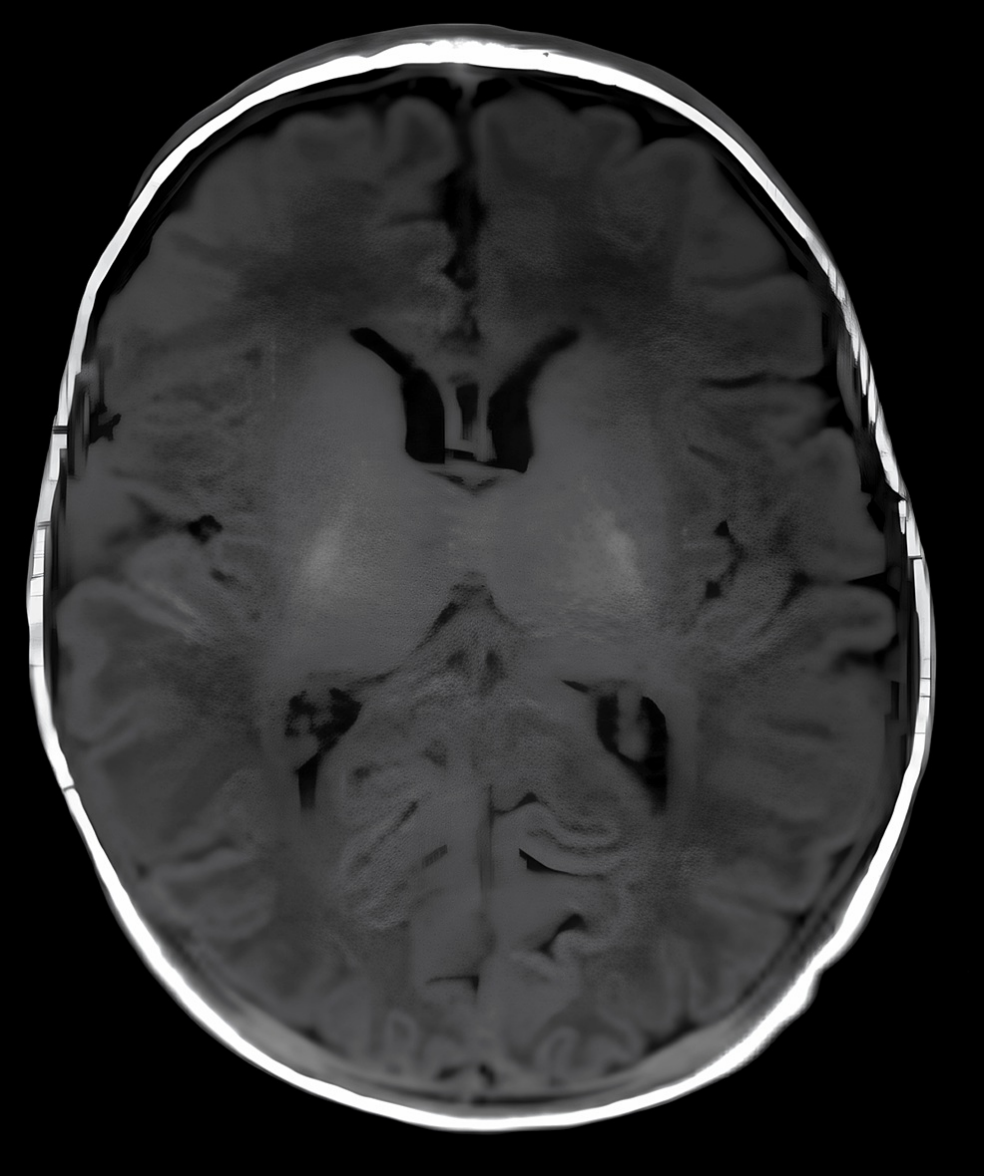
MRI brain at three weeks; T1-weighted image showed a normal structure of the brain. MRI, magnetic resonance imaging

This three-year-old girl of South Asian descent was initially referred to our tertiary care hospital at the age of three weeks with a history of recurrent seizures. She was born by an emergency cesarean section in another facility due to a prolonged second stage of labor. She did not cry soon after birth and needed stimulation and Ambu bagging. The amniotic fluid was meconium-stained, and her Apgar scores were five and six at one and five minutes, respectively, and nine at 10 minutes. Her birth weight was 2.525 kg, and her head circumference was 33 cm. She was shifted to the neonatal intensive care unit (NICU) for observation, and after 12 hours, she was active, pink, and feeding and was handed over to the mother. She had her first seizure around 36 hours of life. The semiology of the seizure included rolling of eyes with tonic posturing. This was associated with apnea, desaturation, and bradycardia. The seizure lasted for around five minutes. She was given midazolam and loaded with phenytoin, and the seizure stopped. The MRI and basic metabolic and infectious workup were normal. Phenytoin was switched to levetiracetam and the patient was discharged. She was then brought to our emergency with status epilepticus with a similar semiology. She was born to non-consanguineous parents, and there was no significant family history of epilepsy. On examination, she did not have any dysmorphic features or neurocutaneous markers. Her neurological examination was unremarkable. The patient was loaded with phenobarbitone in the emergency department but continued to have ongoing seizures and received fosphenytoin, which aborted the seizures. She was admitted to the hospital for further evaluation and management. The metabolic and infectious workup was negative. The interictal electroencephalogram (EEG) and MRI brain were unremarkable (Figure 1). We stopped phenytoin and tried to put her on phenobarbital and clobazam, but then she continued to have seizures until we reloaded her with phenytoin, suggesting that her seizures were responsive to phenytoin.

**Figure 2:**
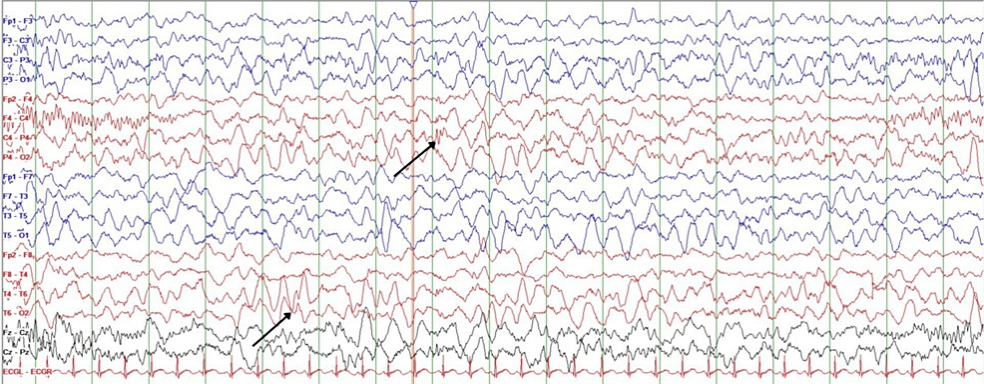
EEG showing right centro-temporal epileptiform discharges. EEG, electroencephalogram

She was discharged on oral phenytoin, clobazam, and levetiracetam. She was seizure-free for the next one month but again presented with status epilepticus. Her seizures were aborted with intravenous fosphenytoin. The phenytoin level sent was low, suggesting that seizure recurrence was due to fluctuation in the phenytoin levels. We assumed a possibility of sodium channelopathy, and an epilepsy gene panel was sent. She was discharged on a combination of phenytoin and oxcarbazepine. The epilepsy gene panel after sequence analysis identified a heterozygous pathogenic variant in FGF12. A heterozygous missense variant in exon 4 of the FGF12 gene (chr3:g.192335434C>T; Depth: 163x) that resulted in the amino acid substitution of histidine for arginine at codon 52 (p.Arg52His; ENST00000445105.7) was detected. This missense change strongly changed the voltage dependence of inactivation gating, resulting in a gain-of-function effect and increased neuronal excitability. Repeat EEG showed occasional right centrotemporal epileptiform discharges (Figure 2). Given the adverse side effect profile of phenytoin, attempts to switch her off from phenytoin to combinations of other antiepileptic medications with sodium channel blocking properties were made without any success. Antiepileptic medications such as valproate, oxcarbazepine, lamotrigine, and lacosamide failed to provide seizure freedom in the absence of phenytoin. She continued to have on and off seizures once in four to five months, mostly when her phenytoin levels were low. The maximum seizure-free period of nine months was obtained on a combination of phenytoin, oxcarbazepine, and lacosamide. She is currently on oxcarbazepine, phenytoin, and topiramate. She did not have any side effects of antiepileptic medication. At present, the patient’s development is progressing well except for mild speech delay.

## Discussion

FGF12 is a part of the FGF family. It encodes proteins necessary for multiple processes in the body such as growth factor as well as transmembrane transporter binding activities, namely for cell/tumor growth, embryogenesis, tissue repair, morphogenesis, etc. It also exhibits a positive regulation of voltage-gated sodium channel activity and increases the excitability of neurons and, thus, is important in the proper functioning and development of the nervous system [1,2].

Mutations in the FGF12 gene, previously referred to as FGF homologous factors 1 (FHF1), are known to be associated with various conditions, including Developmental and Epileptic Encephalopathy 47 (DEE 47), as well as certain cardiac diseases, arrhythmias, pulmonary hypertension, cancers, etc [2,4]. It is an exceptionally rare condition, and affected patients typically present with early-onset seizures, often within the first few days or weeks of life, manifesting in various severities and types [5]. Some present with focal seizures, whereas others have generalized tonic-clonic seizures or tonic seizures [5]. Imagining studies of the brain might show atrophic cerebral changes as well as abnormalities in the white matter. EEG may reveal background slowing accompanied by multifocal epileptic spikes, with or without hypsarrhythmia [5]. The exact mechanism of cerebral atrophy in these cases is still unknown, but research conducted by Siekierska et al. hypothesized that this could be attributed to the hyperexcitotoxic mechanism [6]. In a study by Al-Mehmadi et al., significant MRI findings were reported later in life for four out of five patients. Initially, their MRIs during the early months showed no abnormalities. Among the four patients, three exhibited cerebellar atrophy, while one showed mild prominent cerebellar folia and bilateral mesial temporal sclerosis. All four of these patients had phenytoin in their treatment regimen [7]. 

In a previous case report by Shi et al. in 2017, it was noted that a treatment plan with phenytoin caused the patient to remain seizure-free for a significant amount of time. The patient had seizures that were refractory to different anti-epileptic drugs like clobazam, potassium bromide, phenobarbital, and valproic acid, but once phenytoin (levels at 20 and 35 μg/mL) was added, the seizures stopped. This patient also had MRI findings of mild cerebellar and cerebral atrophy [8].

In one of the largest case series done by Trivisano et al., the majority of the cases were due to de novo mutations in the FGF12 gene, whereas only a few cases had germline somatic or parent mosaicism, shedding light into the prenatal diagnosis of FGF12 mutations in parents with affected children. The seizures in FGF12 mutations were mostly refractory to medications, and they had significant developmental delays. They concluded that antiepileptic drugs (AEDs) targeting sodium channels, such as carbamazepine, lamotrigine, and rufinamide, and most notably, phenytoin, demonstrated the most significant effects. Some other neurological findings included psychomotor regression/delay, intellectual disability, diffuse/limb hypotonia, feeding difficulties, ataxic gait, language disabilities, psychiatric features, autistic features, and/or sleep disturbances. But it is important to remember that like any patients with refractory epilepsy with developmental delays, a multidisciplinary approach to symptomatic management of seizures and developmental delays is of utmost importance. Psychological support and counselling also play a major role [9].

Interestingly, another case series published in 2021 reported a good response to a treatment regimen that included topiramate and valproic acid. It discusses three patients. For the first patient, initially, valproic acid was given to which the patient responded well, but later seizures recurred due to infections so the dose was increased from 15 to 50 mg/kg/day. Levetiracetam was added and discontinued as well, as it did not have any effect on the seizure activity. Finally, topiramate was added, and its dose was also gradually increased over time until the patient was seizure-free at seven months. Their general activities and development improved as the seizure activities were controlled. In the second patient, a combination of levetiracetam along with topiramate could not control the seizures. Later on, their seizures were well controlled with the combination of valproate and topiramate. Similar results were seen in a third patient after a combination of levetiracetam and phenobarbital failed to control seizures [5]. We tried the same combination on our patient as described in that case series but did not observe a favorable response, prompting us to switch back to phenytoin.

Increased seizure activity leads to more regression, as observed in two cases reported by Guella et al. In one instance, a patient treated early with a voltage-gated sodium channel blocker exhibited milder symptoms of regression compared to another patient. The first patient was initiated on phenytoin and topiramate. As seizure activity persisted at a rate of one to three per month, carbamazepine was subsequently added to the medication regimen. Following this adjustment, the patient remained seizure-free for five months, and their initial hypotonia also resolved. The patient continued to progress well, with normal development, EEG, and MRI. Our patient had a similar phenotype and was treated with phenytoin and oxcarbazepine. We achieved good seizure control, with nearly one year of seizure freedom, and the child reached near-normal developmental milestones. However, another patient discussed in the same study still had seizures since the patient did not respond to clonazepam, phenobarbital, lamotrigine, and topiramate. The patient had an allergy to carbamazepine, preventing the administration of phenytoin. Consequently, the final regimen included lamotrigine along with rufinamide, to which the patient responded well. Developmental regression was seen when the frequency of seizures increased. Thus, early control of seizure activity may potentially lead to normal development as seen in these cases [10]. Our experience suggests that early control of seizures leads to a better disease outcome in terms of developmental progression and symptoms.

## Conclusions

Thus, we report a rare case of neonatal onset refractory epilepsy due to FGF12 mutation. The undue favorable response to phenytoin should prompt the physician to consider this condition apart from other sodium channelopathies. After practicing existing literature, our experience prompts us to argue that phenytoin, as opposed to other antiepileptic medications, may be a long-term treatment option in children despite its unfavorable side effect profile, given the positive effects it had on seizure freedom and developmental progression of our patient. A multidisciplinary approach is needed in such cases, along with psychological support to the patient and the family. Further research is needed to look at the exact pathophysiology and formulate a clear treatment guideline for this rare genetic epilepsy.
